# Directed evolution of a cellobiose utilization pathway in *Saccharomyces cerevisiae* by simultaneously engineering multiple proteins

**DOI:** 10.1186/1475-2859-12-61

**Published:** 2013-06-26

**Authors:** Dawn T Eriksen, Pei Chiun Helen Hsieh, Patrick Lynn, Huimin Zhao

**Affiliations:** 1 Department of Chemical and Biomolecular Engineering, Institute for Genomic Biology, University of Illinois-Urbana Champaign, Urbana, IL 61801, USA; 2 Energy Biosciences Institute, Urbana, IL 61801, USA; 3 Department of Molecular and Cellular Biology, University of Illinois at Urbana-Champaign, Urbana, IL 61801, USA; 4 Departments of Chemistry, Biochemistry, and Bioengineering, University of Illinois at Urbana-Champaign, Urbana, IL 61801, USA

**Keywords:** Cellobiose utilization, β-glucosidase, Cellodextrin transporter, Directed evolution, Protein engineering, Pathway engineering, Pathway optimization, Pathway libraries

## Abstract

**Background:**

The optimization of metabolic pathways is critical for efficient and economical production of biofuels and specialty chemicals. One such significant pathway is the cellobiose utilization pathway, identified as a promising route in biomass utilization. Here we describe the optimization of cellobiose consumption and ethanol productivity by simultaneously engineering both proteins of the pathway, the β-glucosidase (gh1-1) and the cellodextrin transporter (cdt-1), in an example of pathway engineering through directed evolution.

**Results:**

The improved pathway was assessed based on the strain specific growth rate on cellobiose, with the final mutant exhibiting a 47% increase over the wild-type pathway. Metabolite analysis of the engineered pathway identified a 49% increase in cellobiose consumption (1.78 to 2.65 g cellobiose/(L · h)) and a 64% increase in ethanol productivity (0.611 to 1.00 g ethanol/(L · h)).

**Conclusions:**

By simultaneously engineering multiple proteins in the pathway, cellobiose utilization in *S. cerevisiae* was improved. This optimization can be generally applied to other metabolic pathways, provided a selection/screening method is available for the desired phenotype. The improved *in vivo* cellobiose utilization demonstrated here could help to decrease the *in vitro* enzyme load in biomass pretreatment, ultimately contributing to a reduction in the high cost of biofuel production.

## Background

The saccharification of lignocellulosic biomass into fermentable sugars is recognized as one of the most expensive operations in biofuel process economics [1,2]. Hydrolysis of the polysaccharide constituents of biomass into simple sugars requires a diverse set of enzymes, namely endoglucanases (EC 3.2.1.4), exoglucanases (EC3.2.1.91), and β-glucosidases (EC 3.2.1.21) [3]. *In vitro* endo- and exoglucanases collectively break down cellulose into cellobiose, a β-1,4-glucose disaccharide. Then, β-glucosidases hydrolyze cellobiose into two glucose monomers, which are subsequently fermented by the cell. To lessen the need of these *in vitro* hydrolytic enzymes, researchers have investigated the use of cellobiose itself as a fermentable sugar [4]. Reducing the price of this *in vitro* enzyme pretreatment would lower the cost of biofuel production and make biofuels more economically competitive with petroleum-derived fuels [1,2].

Three main strategies for heterologous cellobiose utilization have recently been developed. The first strategy involves cell-surface display of extracellular β-glucosidases [4-8]. In this strategy, the cellobiose is hydrolyzed extracellularly and then the glucose is transported into the cell and metabolized. A second strategy, the phosphorolytic pathway, relies on heterologous expression of a cellodextrin transporter and a cellobiose phosphorylase [9-11]. The cellobiose is transported into the cell via the cellodextrin transporter and the intracellular phosphorylase cleaves the disaccharide with an inorganic phosphate, producing a glucose molecule and an α-glucose-1-phosphate, which is quickly metabolized. The hydrolytic pathway is a third strategy, which involves heterologous expression of a cellodextrin transporter and an intracellular β-glucosidase [12-14]. After being transported into the cell by the cellodextrin transporter, the intracellular cellobiose is hydrolyzed by the β-glucosidase into two glucose molecules, which are then metabolized by the cell. Though each strategy has its own advantages, no method is as efficient as glucose assimilation, which is one of the most rapid sugar utilization rates. Therefore, further engineering is required to improve the cellobiose utilization rates to rival glucose consumption. The hydrolytic cellobiose utilization pathway has recently been investigated by our laboratory for optimization through combinatorial transcriptional engineering [15]. Though this method and other transcriptional engineering techniques have been successful in optimizing metabolic pathways [15-18], optimal gene expression cannot overcome the inherent inefficiencies of the proteins within the pathway [19]. Therefore, we sought to apply protein engineering strategies to improve the pathway performance.

Engineering proteins within a pathway has shown to be very successful in improving titer of the desired product [20]. Often these methods tend to isolate the protein from the pathway and engineer it independently from other enzymes within the pathway. In this study, we have investigated a complementary strategy for pathway engineering, wherein we simultaneously modify multiple proteins within the context of the pathway and cellular metabolism. One of the advantages of this strategy is it can identify a mutant pathway with an increased and balanced flux. Engineering pathways with a balanced flux remains a large challenge in metabolic and pathway engineering [17,18,21-25]. By simultaneously screening mutants of all pathway proteins, mutants are not just chosen for a high protein activity, but instead are selected based on an optimized flux and balanced protein activity through the pathway. This method is also advantageous for its simple and efficient library creation which can generate mutations in each protein, allowing for a comprehensive exploration of the potential diversity of the pathway, possibly identifying mutations which could synergistically increase the pathway flux. This approach can be widely applicable to any system where a high-throughput screening/selection method for the desired phenotype is available. The implementation of this directed evolution strategy could further enhance researchers’ abilities to optimize pathways, offering a new avenue for metabolic engineering strategies to include pathway-scale approaches for protein engineering.

**Figure 1:**
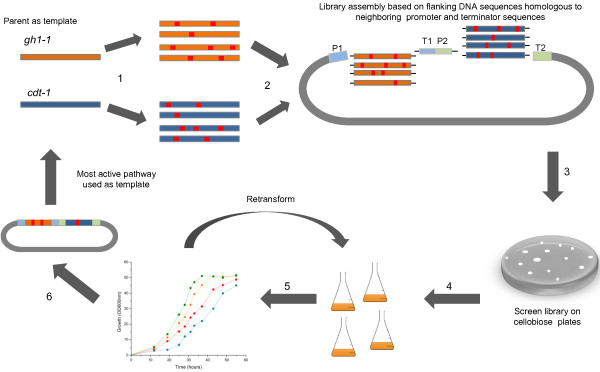
**A general scheme for the directed evolution of multiple proteins within a metabolic pathway.** (**1**) Diversity is introduced to the genes through error-prone PCR, DNA shuffling, or other mutagenesis techniques. (**2**) The genes are then assembled into the pathway through homologous recombination. Each fragment is designed with flanking homologous DNA regions to allow for the recombination. P1 and T1 are the promoter/terminator regions of the first gene, while P2 and T2 are the promoter/terminator for the second gene. No promoter or terminator region was mutagenized. (**3**, **4**) The library is screened based on a high-throughput method to interrogate the phenotype of interest. In this study, screening was accomplished via colony size on cellobiose agar plates. Large colonies are associated with faster growth and chosen for further analysis in flask fermentation. (**5**) To confirm that improved phenotype is a result of the mutated genes of the pathway, the plasmids containing the mutated pathways were isolated and retransformed. (**6**) Improved mutants are chosen for a second round of diversification and screening.

Here we report the first example of simultaneously engineering two proteins, β-glucosidase (gh1-1) [GenBank Accession number XM_951090] and cellodextrin transporter (cdt-1) [GenBank Accession number XM_958708], in *Saccharomyces cerevisiae* for biofuel production (Figure 1). Through directed evolution, key mutations in both proteins synergistically improved the overall cellobiose utilization by 49% and ethanol productivity by 64%. These mutations were directly linked to improved activity or altered substrate specificity of the proteins. In addition to demonstrating improved cellobiose utilization for ethanol production, our results supplement recent research in β-glucosidase substrate specificity studies [26-29] and transporter engineering work [11,30-33].

## Results

### Library construction and screening

**Figure 2:**
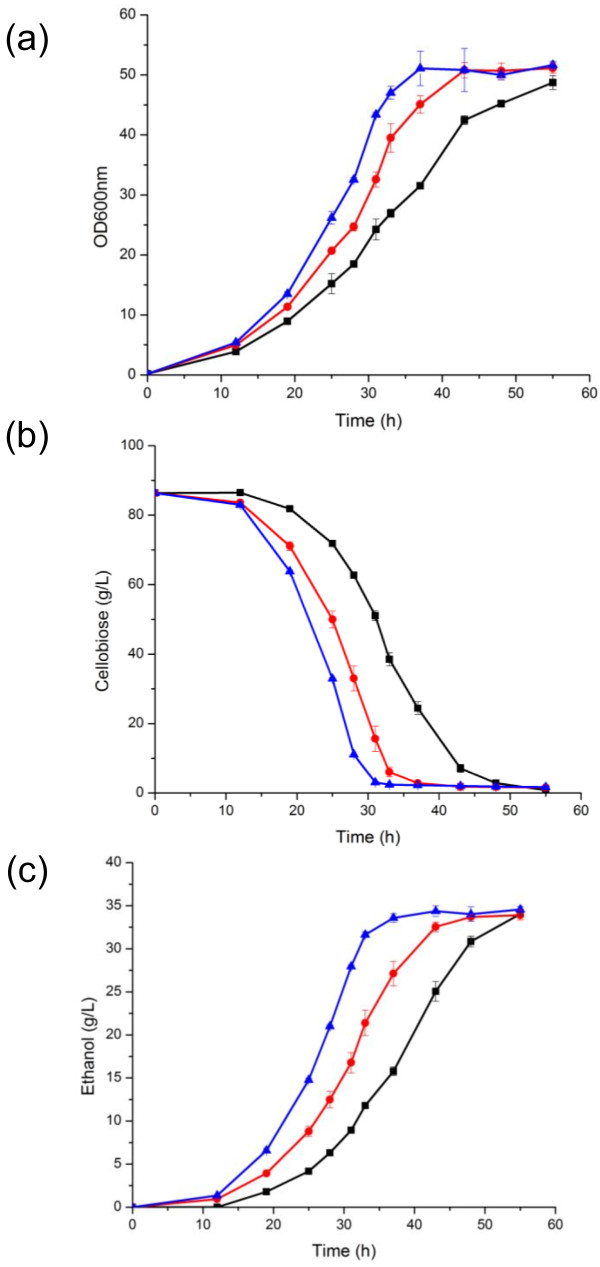
**Fermentation profiles of the cellobiose utilization pathways under oxygen-limited conditions.** The black squares represent the strain harboring the wild-type pathway, the red circle is the strain harboring the R1 pathway and the blue triangle is the strain harboring the R2 pathway. (**a**) Growth curve, (**b**) Cellobiose utilization, (**c**) Ethanol production. Error bars are derived from the standard deviation of biological triplicates.

We created the library of gh1-1 and cdt-1 genes by independently mutagenizing each gene through error-prone PCR. The genes were then co-assembled via DNA Assembler, which utilizes *in vivo* homologous recombination [34], into the single copy plasmid pRS-KanMX, under control of the PYK1 and TEF1 constitutive promoters. The intended mutation rate was one amino acid substitution per protein on average. Considering the combination of both genes, the theoretical total number of mutant pathways was 9.9 × 10^6^[35]. Due to limitations of the transformation efficiency into the industrial yeast host, the actual screened library size was ~10^4^ mutant pathways. The specific growth rate of the strain on cellobiose was used as an assessment of pathway improvement, which allowed for screening to be based on colony size from agar plates [15,36]. Large colonies from the library were visibly distinct from the colonies harboring the wild-type pathway (Additional file 1: Figure S1). The large colonies were picked for quantitative analysis and the strains exhibiting increased specific growth rates on cellobiose were chosen (Additional file 1: Figure S2). These plasmids were extracted and retransformed into fresh yeast cells to confirm that the increased specific growth rate resulted from the mutated pathway, not from genome modification. We successfully screened two rounds of directed evolution, resulting in mutant pathways, R1 and R2, which conferred a higher specific growth rate on cellobiose when compared to the wild-type pathway (Figure 2). A third round of mutagenesis was performed but screening of the library did not result in the identification of a strain with an improved specific growth rate.

### Characterization of the strains expressing the mutant cellobiose utilizing pathways

The first mutant pathway from round one of directed evolution, identified as R1, involved a L173H mutation in the β-glucosidase and a D433G mutation in the cellodextrin transporter. The second round of mutagenesis resulted in pathway R2, with additional mutations: H23L in the β-glucosidase and C82S in the cellodextrin transporter. R1 and R2 pathways conferred increased specific growth rate on cellobiose, improved cellobiose utilization, and higher ethanol productivity compared to the strains harboring the wild-type cellobiose pathway in oxygen-limited conditions (Figure 2). Oxygen-limited conditions were conducted at 100 rpm orbital shaking in un-baffled flasks, which reduced aeration to the culture [37]. There was a 47% increase in specific growth rate in the final mutant strain (0.102 ± 0.002 h^-1^) compared to the wild-type strain (0.0694 ± 0.003 h^-1^). For the metabolite analysis, end-point values at the time required to consume more than 95% of total sugar was used to determine rate, yield, and productivity. The strains harboring the engineered pathways presented a 49% increase in cellobiose consumption and a 64% increase in ethanol productivity (Table 1). Extracellular glucose production (Additional file 1: Figure S3), an undesired byproduct, was produced only when the cellobiose had been almost completely consumed and then was quickly consumed itself. The final strain containing mutant pathway R2 consumed 80 g/L cellobiose in a little less than 30 hours with an ethanol productivity of 1.00 ± 0.03 g/(L · h). To exclude the possibility that the improved phenotype was a result of improved gene expression, we performed quantitative PCR analysis of the mutant and wild-type strains. We also observed the specific protein abundance by monitoring the fluorescence per cell with green fluorescence protein (GFP)-tagging of the proteins. There was no statistically significant difference in mRNA levels or protein abundance between the wild-type and mutant proteins, indicating comparable gene expression levels throughout the strains (Additional file 1: Figure S4).

**Table 1:** Parameters for the strains harboring the improved pathways identified through directed evolution

	**Wild-type**	**R1**	**R2**
Specific Growth Rate	0.0694 ± 0.003	0.0919 ± 0.006	0.102 ± 0.002
(h^-1^ )			
Cellobiose Consumption	1.78 ± 0.06	2.33 ± 0.02	2.65 ± 0.02
(g cellobiose/(L · h))			
Ethanol Productivity	0.611 ± 0.02	0.815 ± 0.03	1.00 ± 0.03
(g ethanol/(L · h))			
Yield			
(g ethanol / g cellobiose)	0.4073 ± 0.02	0.4236 ± 0.0008	0.4363 ± 0.004

*Values determined at time point when >95% cellobiose was consumed for the strain.

Errors are standard deviations of biological triplicates from three independent experiments. Fermentation conditions are in 8% cellobiose rich media and oxygen-limited conditions.

**Figure 3:**
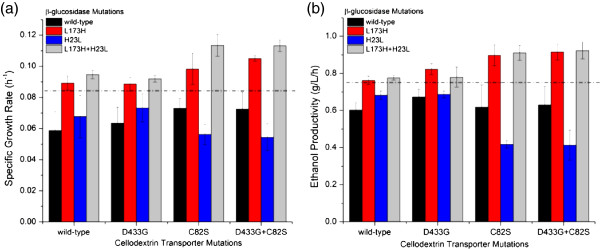
**Pathway analysis to determine how each mutation contributes to the improved phenotype.** The graphs depict the (**a**) growth rate and (**b**) ethanol productivity of the strains containing the indicated individual and combined mutants of the pathway grown on cellobiose. Each round of directed evolution discovered a mutation in both proteins and these data depict how individual mutations from each round of evolution contributes to the phenotype of the strain harboring the mutant pathway. The x-axis represents the individual and combined mutations of the cellodextrin transporter and each bar depicts the individual and combined mutations of the corresponding β-glucosidase enzyme of the strain. The black bar is wild-type β-glucosidase, the red bar is is L173H mutant, the blue bar represents the H23L mutant, and the grey bar is the double mutant L173H + H23L. The dotted lined denotes values above which are statistically significant over the wild-type (*p* < 0.05) as determined through a student’s t-test. Error bars represent the standard deviation of biological triplicates.

To determine which mutations conferred the increased specific growth rate and ethanol productivity, the individual and combined mutants from each round were analyzed (Figure 3). The most significant mutants for the phenotype improvements of the strains were shown to be the L173H mutation in the β-glucosidase and the C82S mutation in the cellodextrin transporter. Specifically, the L173H mutation was attributed to increased specific growth rate, while the C82S mutation increased both the growth rate and ethanol productivity.

### Activity assays

#### β-glucosidase assay

The enzymes were assayed *in vitro* to better identify how the mutations affected the activity of the individual enzymes. We assayed the activity towards cellobiose by enzymatically quantifying the glucose produced by a timed reaction of β-glucosidase with cellobiose. The L173H mutant increased the specific activity towards cellobiose by nearly 90% compared to the wild-type. The single H23L mutant had only slightly higher specific activity than the wild-type enzyme. The β-glucosidase combined mutant L173H and H23L increased cellobiose hydrolytic activity by over 150% compared to the wild-type enzyme, illustrating additive effects from the combined mutations (Table 2). Further tests involved *p*-nitrophenyl β-D-glucopyranoside (*p*-NPG), a synthetic substrate commonly used for determining β-glucosidase activity [26,28]. Compared to the wild-type, the L173H mutant decreased hydrolytic activity towards *p*-NPG by 34%. The single mutant H23L protein had activity slightly higher than wild-type. The combination of the L173H and H23L mutations increased the activity to be slightly higher than the single L173H mutation.

**Table 2:** Specific activity measured from the crude lysate of the engineered β-glucosidase

	***p*-NGP**	**Cellobiose**
	**(U/mg protein)**	**(U/mg protein)**
Control	0.00729 ± 0.0001	0.0021 ± 0.002
Wild-type	0.193 ± 0.01	0.0668 ± 0.001
L173H	0.130 ± 0.01	0.124 ± 0.004
H23L	0.219 ± 0.004	0.0738 ± 0.006
L173H + H23L	0.156 ± 0.007	0.170 ± 0.01

One unit is defined as one μmol/min and the control is the lysate from a strain with no heterologously expressed β-glucosidase. Errors are derived from the standard deviation of biological triplicates.

#### Cellodextrin assay

The specific activity of the cellodextrin transporter was measured through a modified oil-stop assay based on the rate of radiolabeled sugar uptake [12,38]. The single mutation D433G conferred an 18% increase in cellodextrin transporter activity. When compared to wild-type, the single mutant protein containing C82S increased the transporter activity by 48%. The combination of D433G and C82S mutations increased the protein activity over wild-type by 60% (Table 3).

**Table 3:** Specific activity measurements from the cellodextrin transporter as determined through radioactive-labeled cellobiose uptake rate assay

	**Cellobiose uptake rate**
	**(U/gcdw)**
Control	0.006 ± 0.001
Wild-type	1.77 ± 0.07
D433G	2.09 ± 0.05
C82S	2.63 ± 0.2
D433G + C82S	2.82 ± 0.2

One unit is defined as one μmol/min. The control is the lysate from a strain with no heterologously expressed cellodextrin transporter. Errors represent the standard deviation of biological triplicates.

### Structural modeling

**Figure 4:**
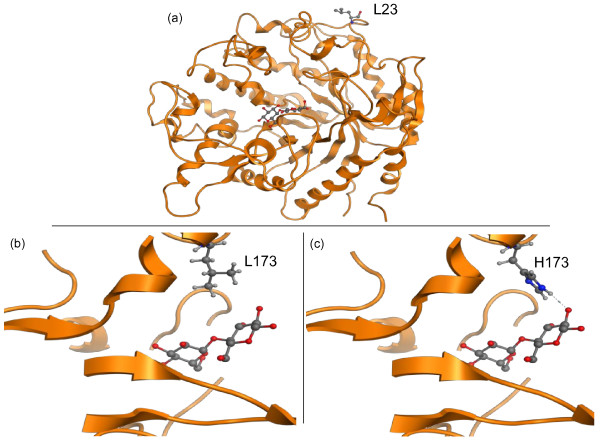
**Homology model of the *N. crassa *β-glucosidase with the cellobiose substrate docked into the active site.** (**a**) The full β-glucosidase homology model illustrating the L23 mutation far from the active site. (**b**) There are no predicted ligand interactions of the cellobiose with the wild-type L173 residue within the active site. (**c**) The mutated residue H173 is predicted to have a direct hydrogen bond with the hydroxyl group of the C1 atom of the cellobiose molecule.

A homology model was constructed for the β-glucosidase and the mutations were investigated for possible structure-function relationships. The H23L mutation is on the periphery of the enzyme, far from the active site (Figure 4a). The homology model predicted that the L173H mutation was located within the active site, in a domain which has previously been identified as the substrate entrance region [27]. The amino acid L173 did not have any predicted interactions with cellobiose (Figure 4b). When the residue was mutated to H173, the model predicted a direct hydrogen bond to the hydroxyl group of the C1 atom of the cellobiose (Figure 4c).

**Figure 5:**
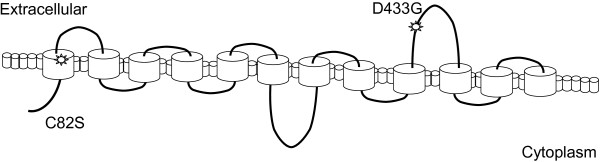
**Sequence-structure based mapping of the cellodextrin mutations.** The HMMTOP software predicts sequence-structure of the transmembrane helix, inner, and outer loops of transmembrane proteins. The mutations of the cellodextrin transporter discovered from this study are overlaid on the structure prediction from the HMMTOP software.

Since no crystal structure is available for the cellodextrin transporter, to visualize and hypothesize how these mutations affect the protein, a sequence-based analysis was performed with the aid of HMMTOP software [39,40]. This software is used to predict transmembrane helix domains, helix tail domains, and extra/intracellular loop structures (Figure 5). Based on the predicted structure, the cellodextrin transporter is comprised of 12 transmembrane helices with one large inside loop and one large outside loop. The D433G mutation is predicted to be located on the large outside loop and the C82S mutation is predicted to be in the first transmembrane helix.

## Discussion

Efficient production of biofuels and specialty chemicals hinges on optimization of the metabolic pathways associated with the desired product. In this endeavor, a variety of successful pathway optimization methods, mainly based on transcriptional engineering, have been developed [15-18,41-44]. Though successful, these methods are unable to overcome inefficiencies based on innate enzyme characteristics such as V_max_ and K_m_, therefore protein engineering strategies must also be applied [19]. To improve these properties, often the enzymes are removed from the pathway context and engineered *in vitro* or *in vivo* for the highest activity. Engineering each enzyme independently can be arduous and there is no guarantee the engineered enzyme will reduce the bottleneck when it is reinstated into the pathway. We are introducing a new approach to combine pathway and protein engineering, involving the evolution of all proteins within the pathway simultaneously. Through this strategy, no *a priori* information about pathway bottlenecks or enzymes is required for the directed evolution. Mutant proteins of the pathway are not chosen for high activity, but instead interrogated as a whole for a balanced, increased activity and flux which is demonstrated by an improved pathway phenotype. This combinatorial mutagenesis library strategy allows for a comprehensive exploration of the pathway potential diversity, which could identify unexpected synergistic effects [42,45]. Recent advances in large-scale library creation with high efficiency have made the multi-protein library a possibility [15,36,46-48]. Our study illustrates an *in vivo* library creation for simultaneous engineering of multiple proteins in a pathway through directed evolution in *S. cerevisiae*. To the best of our knowledge, there has been only one example of mutagenesis of multiple proteins through directed evolution, which was illustrated in *Escherichia coli* for arsenate resistance [49]. This strategy has not been widely implemented and has not been applied to other systems. Our work optimizes a sugar utilization pathway for increased metabolite production with a simple one-step assembly method, allowing for quick and efficient rounds of evolution. This represents the first example of pathway optimization through multiple protein engineering in *S. cerevisiae*.

The final optimized pathway (R2) increased cellobiose consumption by 49% and ethanol productivity by 64%. Characterization of the strains expressing the mutations which conferred these improved phenotypes illustrated that the highest specific growth rates on cellobiose were achieved only when the β-glucosidase L173H mutation is present. In the cellodextrin transporter, the most significant mutation was the C82S mutation. It was shown that the optimized pathway involved an overall increase of protein activity: the β-glucosidase had an increased specificity towards cellobiose and the cellodextrin transporter exhibited a higher overall activity.

The β-glucosidase from glycoside hydrolase family 1 (GH1) contains the standard (α/β)-barrel structure of the enzyme family. The active site is located in a deep cleft formed by the connection loops at the C-terminus of the β-sheets of the TIM barrel with negatively charged residues surrounding the bottom of the active site [28]. The conserved catalytic residues of the GH1 family involve the catalytic acid/base Glu 166 and the nucleophile Glu 377, which cleave the glycosidic bond via the common double displacement mechanism [26]. This mechanism acts on β-1,4-linked glucose derivatives; the enzyme is promiscuous, being active towards a range of substrates with varying affinities [28]. In this study, the wild-type GH1-1 β-glucosidase enzyme exhibited an innate substrate preference for *p*-NPG over cellobiose. The mutation L173H shifted the substrate specificity towards cellobiose. Homology modeling suggested the H173 residue could have a direct hydrogen bond with the hydroxyl group of the C1 atom of the cellobiose (Figure 4c), which was not predicted with the wild-type β-glucosidase. It is possible that this new hydrogen bond could increase the affinity of the enzyme for cellobiose by firmly keeping the substrate in place during hydrolysis, a hypothesis previously established by studies in substrate recognition for the β-glucosidase family [26,29]. There has been significant interest in substrate specificity of the GH1 family, which is suggested to occur via an extensive network of polar interactions with well-ordered water molecules and enzyme-substrate bonding [26-29]. A specific study of interest involved a β-glucosidase from *Trichoderma reesei* [GenBank Accession number AB003110.1] which was recently investigated to identify critical residues within the substrate entrance region [27]. Through rational design, substitutions at residue 172 were shown to improve substrate recognition, thermostability, and enzyme activity. This β-glucosidase has a 73% sequence identify to the GH1-1 enzyme studied here. Sequence alignment and structural modeling predicted this residue to be in the same position as the L173 residue from this work, corroborating the importance of this residue position. The H23L mutation is hypothesized to be an overall activity enhancer, with only slightly improved enzyme activity towards both substrates *p*-NPG and cellobiose. H23 is located on the periphery of the enzyme, distant from the active site (Figure 4a).

Engineering sugar transporters has recently been shown to greatly enhance pathway productivity [11,30], a result also illustrated here. In our directed evolution effort, the combined D433G and C82S mutations of the cellodextrin transporter increased the specific activity by 60% compared to the wild-type. The specific activity is directly proportional to the V_max_ of the transporters, which is the protein property that has been most often enhanced by sugar transporter engineering [11,30]. It is probable that this property is the most likely to be improved due to the screening conditions, which were performed at saturating conditions. The cellodextrin transporter model predicted that the D433G mutation is located on a large outside loop. It is possible that this loop could have been causing a steric hindrance for the mass transfer of cellodextrin to the transporter, which could have been alleviated by the altered loop structure associated with this mutation. The C82S mutation, which conferred the highest increase in activity, is predicted to be located in the first transmembrane helix (Figure 5). The location in the helix and the increased activity suggest that this mutation could be directly associated with cellobiose interactions or the protein complex stability. Young et al. investigated directed evolution of xylose transporters and identified mutations which tended to cluster around the first transmembrane helix [30], a similar finding to this study.

## Conclusions

Cellobiose utilization has recently become a significant consideration in economical biofuel production. Efficient utilization could decrease the expensive *in vitro* enzyme load in biomass pretreatment. We successfully optimized the hydrolytic cellobiose utilization pathway through a new directed evolution strategy: simultaneously engineering multiple proteins within the context of the pathway. The optimized pathway involved a β-glucosidase mutant with an increased specificity towards cellobiose and a cellodextrin transporter mutant with an increased overall activity. By applying directed evolution to the entire pathway, engineered proteins can be found which synergistically improve the phenotype either through a balanced flux of the pathway or through overall protein activity improvement. Simultaneous multi-protein engineering is expected to expand researchers’ abilities to optimize biosynthetic metabolic pathways.

## Materials and methods

### Strains, media, and culture conditions

The industrial *Saccharomyces cerevisiae* strain Still Spirits (Classic) Turbo Distiller’s Yeast was purchased from Homebrew Heaven (Everett, WA). Yeast strains were cultivated in YP media (1% yeast extract, 2% peptone) with 2% glucose (YPD) or 2-8% cellobiose (YPC). YPC with 8% cellobiose was used in fermentation analysis while 2% cellobiose was used for plate screening. *S. cerevisiae* strains were cultured at 30°C with orbital shaking at 250 rpm for aerobic growth or 100 rpm for oxygen-limited conditions in non-baffled flasks. As needed, 200 μg/mL G418 (KSE Scientific, Durham, NC) supplemented the YPD for pRS-KanMX plasmid selection. *Escherichia coli* DH5α (Cell Media Facility, University of Illinois at Urbana-Champaign, Urbana, IL) was used for recombinant DNA manipulations. *E. coli* strains were cultured in Luria broth (LB) (Fischer Scientific, Pittsburgh, PA) at 37°C and 250 rpm, supplemented with 100 μg/mL ampicillin. Yeast and bacterial strains were stored in 15% glycerol at −80°C. All chemicals were purchased from Sigma Aldrich or Fisher Scientific.

### Plasmid and strain construction

Restriction enzymes were purchased from New England Biolabs (Ipswich, MA). All cloning work was performed via DNA Assembler, which relies on the *in vivo* homologous recombination mechanism in yeast [34]. Yeast plasmids were isolated using Zymoprep Yeast Plasmid Miniprep II kit (Zymo Research, Irvine, CA), then transformed into *E. coli* for isolation of high purity DNA. *E. coli* plasmids were isolated using Qiagen Spin Plasmid Mini-Prep Kit (Qiagen, Valencia, CA). PCR fragments were purified by Qiagen QIAQuick Gel Purification Kit.

The β-glucosidase gene gh1-1 [GenBank Accession number XM_951090] from *Neurospora crassa* was expressed using a PYK1 promoter and an ADH1 terminator. The cellobiose transporter gene cdt-1 [GenBank Accession number XM_958708] from *N. crassa* was expressed using a TEF1 promoter and a PGK1 terminator as previously constructed [14]. To transfer the pathway to the pRS-KanMX plasmid [15], primers kanMX-PYKp-F and PGKt-kanMX-R (Additional file 1: Table S1) were designed to amplify the full cellobiose utilization pathway. To facilitate the creation of a library of cellobiose utilization pathways, a pRS-KanMX helper plasmid was constructed. The helper plasmid contained the PYK1 promoter and the PGK1 terminator, separated by a unique restriction enzyme recognition site *Bam*HI for plasmid linearization. The helper plasmid was later linearized and used as a backbone for the library assembly. The PYK1 promoter was amplified using primers kanMX-PYKp-F and PYKp-BamHI-PGKt-R (Additional file 1: Table S1). The PGK1 terminator was amplified using primers PYKp-BamHI-PGKt-F and PGKt-kanMX-R (Additional file 1: Table S1).

### Library creation

The ADH1 terminator and TEF1 promoter were not subjected to random mutagenesis, thus this cassette was amplified independently using primers internal-BGL-ADHt-F and internal-TEFp-CDT-R (Additional file 1: Table S1) by standard PCR amplification. The gh1-1 and cdt-1 genes were subjected to error prone PCR with error rates of 2–3 basepair mutations per gene, resulting in one amino acid mutations per protein on average. Each fragment was amplified with 60–80 basepair homologies to upstream and downstream DNA sequences. The three fragments were co-transformed with the linearized helper plasmid into *S. cerevisiae*. The library size obtained was 10^4^, consistent with libraries previously generated by this method [15,36]. To confirm diversity of the library, ten colonies were randomly picked from the YPD + G418 plate and their plasmids were isolated and sequenced.

### Library screening

The library was screened on YPC plates for large colonies [15,36] (Additional file 1: Figure S1). The growth rate of the largest colonies was quantified by small-scale fermentation. The colonies were seeded overnight in YPD + G418, washed twice with sterile water, and then inoculated into 10 mL YPC unbaffled flasks at 100 rpm and 30°C (Additional file 1: Figure S2). After confirmation of the top 5 clones with the fastest growth rate, the plasmids were isolated and retransformed. After retransformation, the strains were seeded overnight in YPD + G418, washed twice with sterile water, and then inoculated into 50 mL YPC in 250 mL unbaffled flasks at 100 rpm and 30°C. The cultures were sampled and analyzed for growth and metabolite production as described previously [15,36]. After confirmation of the strains with improved phenotype, the plasmids were isolated and sequenced to identify the mutations within the genes of the pathway. The genes were used as a template for a second round of directed evolution.

### Fermentation analysis

Seed cultures were inoculated from plates of freshly streaked frozen culture stocks. YPD + G418 seed cultures were used for the individual and combined mutant pathway fermentation tests. YPC seed cultures were used for final mutant pathway analysis. The seed cultures were grown at 30°C and 250 rpm overnight, then washed with sterile water twice before inoculating 50 mL YPC in 250 mL un-baffled flasks to an initial OD of 0.2. The cultures were incubated at 30°C and 100 rpm orbital shaking for oxygen-limited growth [37]. The cultures were sampled and analyzed for growth and metabolite production as previously described [15,36]. For the metabolite analysis, end-point values at the time required to consume more than 95% of total sugar was used to determine rate, yield, and productivity.

### Construction of single mutants

The single mutant genes were constructed through site-directed mutagenesis and the mega primer PCR method with primers listed in Additional file 1: Table S1. After PCR amplification, the genes were transformed into the Classic strain along with the ADHt1/TEFp1 cassette into a linearized pRS-KanMX helper plasmid. The final plasmid was purified and sequenced for confirmation.

### β-Glucosidase enzyme activity assays

Classic strains harboring an empty vector, the wild-type, and mutant pathways were grown to mid-exponential phase and washed three times with potassium phosphate buffer (pH 7). A final cell mass equivalent to an OD600 of 20 was harvested. Cell-free extracts were prepared through YPER Extraction Reagent (Thermo Scientific, Rockport, IL), 125 μL of YPER was used to lyse the cells for 20 minutes at 25°C with vigorous shaking at 700 rpm in a thermomixer (Eppendorf, Germany). After lysing, the cell debris was pelleted for 10 minutes with 15,000 rpm at 4°C. The total protein concentration was determined via the BCA protein assay kit (Pierce, Rockford, IL), the standard manufacturer protocol was followed.

The lysate was tested for *p*-NPG activity with 1 mM *p*-NPG in 100 mM potassium phosphate buffer at pH 7. The colorimetric change was monitored at 405 nm in a 96-well Biotech Synergy 2 plate-reader (Winooski, VT) for 30 minutes at 30°C. The amount of p-nitrophenol (p-NP) released was quantified by a p-NP standard in100 mM potassium phosphate buffer pH 7. One unit of activity is equivalent to 1 μmol of p-NP released per min. For cellobiose-based enzymatic assay, the linear range of the β-glucosidase was determined. The reactions were carried out at 60 mM cellobiose in 100 mM potassium phosphate buffer pH 7. After addition of lysate, the reaction was allowed to react for 15, 30, 45, and 60 minutes at 30°C. The reaction was stopped by boiling at 100°C for 10 minutes. The samples were then centrifuged at 15,000 rpm for 5 minutes before being stored on ice. The amount of glucose which had been produced in the allotted time frame was then measured using the D-glucose kit (R-Biopharm, Germany). Standard manufacturer’s instructions were followed. To determine the β-glucosidase protein abundance in each strain, the wild-type and mutant proteins were fused to GFP. There was no statistically significant difference in β-glucosidase abundance of each strain (Additional file 1: Figure S4). The total protein concentration, as determined through BCA assay, was comparable in each lysate. Therefore, the ratio of β-glucosidase to total protein was assumed constant, and hence the activity was normalized to total protein concentration. One unit (U) is defined as one micromole of glucose produced per minute.

### CDT activity assay

The cellodextrin transporter was assayed using the oil-stop protocol previously reported [12,38]. Cultures of the wild-type and mutant strains were grown in YPC to an OD of 15–20, washed three times with ice-cold assay buffer (30 mM MES-NaOH + 50 mM ethanol), and then normalized to an OD of 20. 50 μL of cells were added to 50 μL of [^3^H]-cellobiose (Movarek Biochemicals, Brea, CA) at 30°C and layered over 100 μL of silicone oil (Sigma 85419), incubated for 10, 20, 40, and 80 seconds. The cells were then centrifuged through the oil at 15,000 rpm for 30 seconds. After being placed in ethanol/dry-ice bath, the cell pellets were solubilized in NaOH overnight. The amount of [^3^H]-cellobiose present in the cells was then quantified by a Beckman Coulter LS 6500 liquid scintillation counter (Brea, CA). The amount of labeled cellobiose that was transported into the cell was plotted against the time of reaction. One unit (U) is defined as one micromole of cellobiose taken up by the cell per min. The rate of the cellobiose uptake was normalized by the transporter abundance determined through GFP fluorescence measurements (Additional file 1: Figure S4) and the gram cell dry weight (gcdw).

### Homology modeling

A homology model of the β-glucosidase enzyme was constructed to identify the structure-function relationships of the mutations discovered. The gene encoding the β-glucosidase from *Trichoderma reesei*[28] [PDB accession code 3AHY] afforded the highest homology to the gh1-1 gene from *N. crassa* with 73% sequence identity and was used as a template for homology modeling. The structure model of the β-glucosidase from *N. crassa* was constructed using the modeling program Molecular Operating Environment (Chemical Computing Group, Montreal, Canada). After constructing the homology model, the substrate in the co-crystal structure of *Neotermes koshunensis* β-glucosidase [PDB accession code 3VIK] was docked into the model [27]. The model was energy minimized before ligand interactions were investigated. To identify the effects of the mutations, the mutations were introduced to the model and the energy was minimized again before investigating the ligand interactions.

## Abbreviations

p-NPG: p-nitrophenyl β-D-glucopyranoside; p-NP: -nitrophenol; gcdw: Gram cell dry weight.

## Competing interests

The authors declare there are no competing interests.

## Authors’ contributions

DTE and HZ designed the study, analyzed data, and wrote the manuscript. DTE, PCHH, and PL performed the experiments. All authors have read and approved the final manuscript.

## Supplementary Material

Additional file 1: Table S1 Primers used in this study. **Figure S1.** Selection plates of the error-prone library on cellobiose plates, depicting the large colonies. **Figure S2.** Flask screening data before retransformation of top clones from the first round of error-prone PCR. **Figure S3.** Flask screening data before retransformation of top clones from the first round of error-prone PCR. **Figure S4.** Consistent protein expression levels between mutants. Click here for file
