# Supplementary material for: Directed evolution of a cellobiose utilization pathway in Saccharomyces cerevisiae by simultaneously engineering multiple proteins
Source: Microb Cell Fact. 2013 Jun 26;12:61. doi: 10.1186/1475-2859-12-61 (PMC3702475; doi:10.1186/1475-2859-12-61)

# Directed evolution of a cellobiose utilization pathway in *Saccharomyces cerevisiae* by simultaneously engineering multiple proteins

Dawn T. Eriksen^1^, Pei Chiun Helen Hsieh^1^, Patrick Lynn^2^, Huimin Zhao^1,2,3*^

^1^Department of Chemical and Biomolecular Engineering, Institute for Genomic Biology,

^2^Department of Molecular and Cellular Biology, University of Illinois at Urbana-Champaign, Urbana, IL 61801

^3^Departments of Chemistry, Biochemistry, and Bioengineering, University of Illinois at Urbana-Champaign, Urbana, IL 61801

**Additional File 1**

* To whom correspondence should be addressed. Phone: (217) 333-2631. Fax: (217) 333-5052. E-mail: zhao5@illinois.edu.

**Additional Materials and Methods**

**qPCR analysis**

Samples of cultures grown in the same conditions as protein activity assays, beginning-mid exponential phase, and were used to compare relative mRNA expression levels of the mutant pathways via quantitative PCR (qPCR). The total RNA was isolated from fresh samples using the yeast FastRNA SPIN kit (MP Biomedical Solon, OH) following manufacturer’s instructions. Isolated RNA samples were cleaned using TURBO DNA-free Kit (AMBION INC, Austin, TX) and then reverse transcribed into cDNA using the Transcriptor First Strand cDNA Synthesis kit (Roche, Mannheim, Germany) with the oligo-dT primer following the manufacturer’s instructions. The qPCR was performed with LightCycler 480 SYBR Green Master reagents (Roche) using the Roche Light Cycler^®^ 480 System (Roche, Indianapolis, IN). The relative abundance of the mRNA levels for the target genes was normalized to the asparagine-linked glycosylation 9 gene (*alg9*) expression to determine expression levels.

**GFP fusion**

The β-glucosidase and the cellodextrin transporter genes were C-terminally tagged with GFP, using (Gly-Gly-Gly-Gly-Ser)_2_ linker, using primers described in Table S1 and assembled via DNA Assembler. Strains harboring the GFP-tagged proteins were grown in the same conditions as the protein activity assays: inoculated to an initial OD of 0.2 and grown at 30 °C at 100 rpm orbital shaking until the cells reached OD 15-20. The cells were then diluted in PBS to an OD of about 0.1-0.2 and were analyzed on a flow cytometer (Biosciences LSR II Flow Cytometry Analyzer, BD Biosciences, San Jose, CA) to quantify GFP fluorescence of each mutant protein in the strain, with excitation wavelengths at 488 nm and emission band pass 550/30.

**Table S1:** Primers used in this study

| PGKt-kanMX-R | 5’-CCTCACTAAAGGGAACAAAAGCTGGAGCTCCACCGCGGTGGCAGGAAGAATACACTATAC-3’ |
| --- | --- |
| BGL EP-F | 5’-CTCTCTTGTTTCTATTTACAAGACACCAATCAAAACAAATAAAACATCATCACAATG-3’ |
| BGL EP-R | 5’-GCCGACAACCTTGATTGGAGACTTGACCAAACCTCTGGCGAAGAAGTCCAAAGCTTTA-3’ |
| CDT EP- F | 5’-CTTCTTGCTCATTAGAAAGAAAGCATAGCAATCTAATCTAAGTTTTAATTACAAAATG-3’ |
| CDT EP-R | 5’-GGATGGGGAAAGAGAAAAGAAAAAAATTGATCTATCGATTTCAATTCAATTCAATCTA-3’ |
| internal-BGL-ADHt-F | 5’-GAAGCCGCTCTTTGACTCTTTGATCAAGAAGGACTAAAGCTTTGGACTTCTTCGCCAGAG-3’ |
| internal-TEFp-CDT-R | 5’-CGGTGCTGGCCCCGTCATGGGAGCCGTGAGACGACATTTTGTAATTAAAACTTAGATTAG-3’ |
| B2-H23L-F | 5’-GGGTGCTATCCTCGCCGACGGCCGTGGC-3’ |
| B2-H23L-R | 5’-GCCACGGCCGTCGGCGAGGATAGCACCC-3’ |
| C2-C82S-F | 5’-CGCCTTTTGTAGTGCATGCGCCAACGG-3’ |
| C2-C82S-F | 5’-CCGTTGGCGCATGCACTACAAAAGGCG-3’ |
| qPCR-BGL-F | 5’-AACCAGAAGGGCATCGACCACTAT-3’ |
| qPCR-BGL-R | 5’-GCACTTGGGAATGGCCTTGAACAT-3’ |
| qPCR-CDT-F | 5’-AATCCCCTCGCTTCCTATTTG-3’ |
| qPCR-CDT-R | 5’-TCCTGATACCGTCCCTCATC-3’ |
| CDT sequencing-TEPp-F | 5’-CTTTCGATGACCTCCCATTGATATTTAAG-3’ |
| CDT sequencing- PGKt-R | 5’-CGAATGGGAAAAAAAAACTGCATAAAGGC-3’ |
| BGL sequencing-F | 5’-CCTTTGGTTTTTTATTCTTAACTTG-3’ |
| BGL sequencing- R | 5’-GAAGTGTCAACAACGTATCTACCAACG-3’ |
| Prs Kanmx-PYK-BGL-F | 5’-CGACGTTGTAAAACGACGGCCAGTGAGCGCGCGTAATACGAATGCTACTATTTTGGAG-3’ |
| BGL-Linker+GFP-R | 5’-CTCATAGAACCTCCACCTCCAGAACCTCCACCTCCGTCCTTCTTGATCAAAGAGTC-3’ |
| BGL-Linker+GFP-F | 5’GAGCTTGAAGCCGCTCTTTGACTCTTTGATCAAGAAGGACGGAGGTGGAGGTTCTGGAGGTGGAGGTTCTATGAGTAAAGGAGAAGAACTTTTCACTGG-3’ |
| Linker+GFP-AHDt-R | 5’-GGAGACTTGACCAAACCTCTGGCGAAGAAGTCCAAAGCTCTATTTGTATAGTTCATCC-3’ |
| Linker+GFP-ADHt-F | 5’-GCTGGGATTACACATGGCATGGATGAACTATACAAATAGAGCTTTGGACTTCTTCGCCAG-3’ |
| ADHt-CDT Cas-KanMX-R | 5’-CCTCACTAAAGGGAACAAAAGCTGGAGCTCCACCGCGGTGGCAGGAAGAATACACTATAC-3’ |
| BGL CDT Cassette-Linker GFP-R | 5’-CTTTACTCATAGAACCTCCACCTCCAGAACCTCCACCTCCAGCAACGATAGCTTCGGAC-3’ |
| BGL CDT- Linker GFP-F | 5’CGTCCAGGCCGACGGCCATGTGTCCGAAGCTATCGTTGCTGGAGGTGGAGGTTCTGGAGGTGGAGGTTCTATGAGTAAAGGAGAAGAACTTTTCACTGG-3’ |
| Linker GFP- PGKt-R | 5’-GAAAAAAATTGATCTATCGATTTCAATTCAATTCAATCTATTTGTATAGTTCATCC-3’ |
| Linker GFP-PGKt-f | 5’-GCTGGGATTACACATGGCATGGATGAACTATACAAATAGATTGAATTGAATTGAAATCG-3’ |

**Figure S1:** Selection plates of the error-prone library on cellobiose plates, depicting the large colonies. (a) Wild-type pathway with all uniform colonies, (b) error-prone PCR library.

(a) (b)

(a) (b)

**Figure S2:** Flask screening data before retransformation of top clones from the first round of error-prone PCR. Plates were screened for the top 200 biggest colonies. These colonies were then selected for tube screening. The top 10% fastest growers from the tube-screen were retested in flasks with results shown here. (a) Specific growth rates of these top 10% mutants: (■) is the wild-type pathway; (▲) represent the growth rate of the top 10% fastest growers from the tube initial screen; (●) depicts the clone 5, which was selected as R1. The dotted line represents the cut-off for specific growth rates of clones selected for further analysis and retransformation. (b) Cellobiose utilization of the top 10% mutants before retransformation: (■) represents the wild-type pathway, (●) depicts the clone 5, which was selected as R1. Other clones in this graph with significant cellobiose consumption did not exhibit this increased phenotype after retransformation, while clone 5 activity was consistent after retransformation.


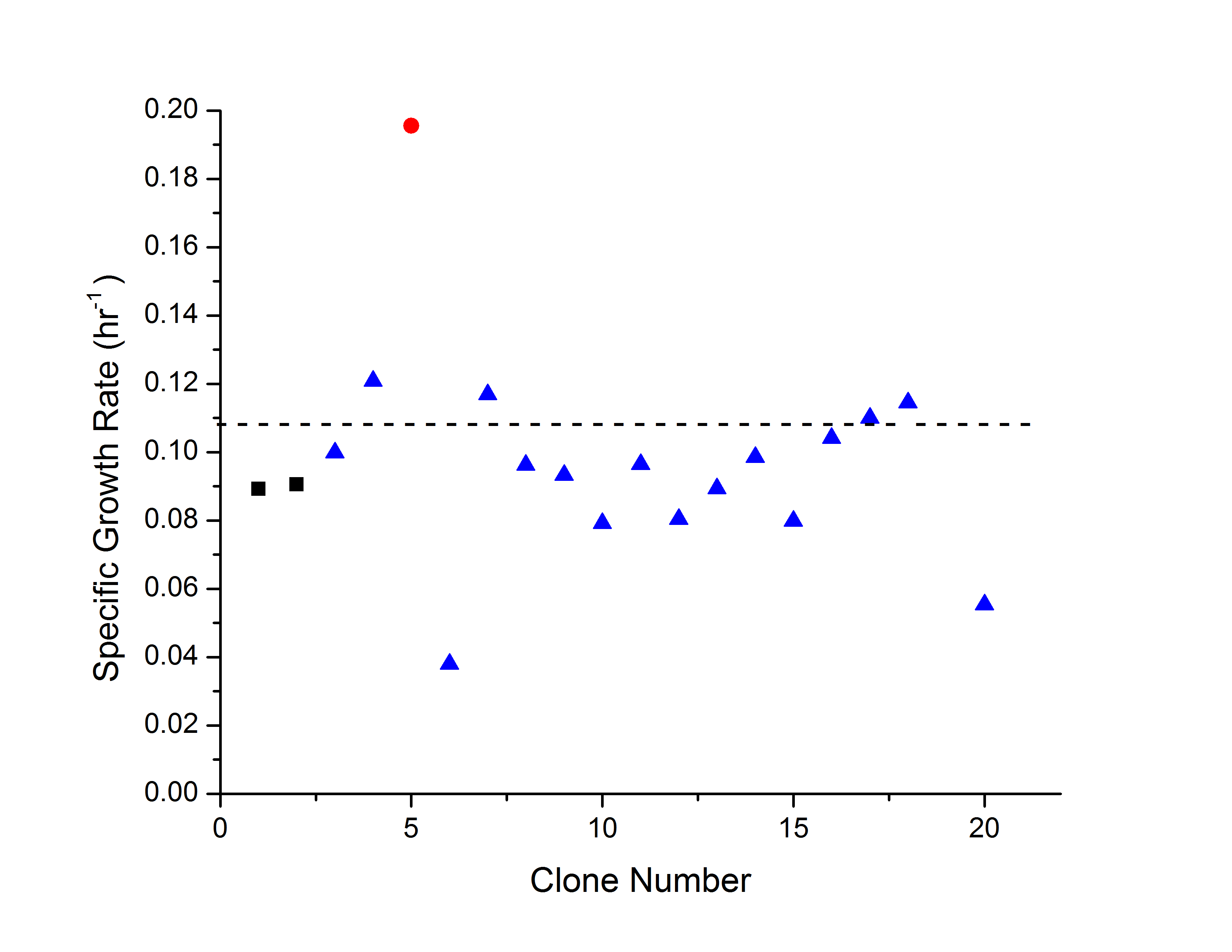


(a)


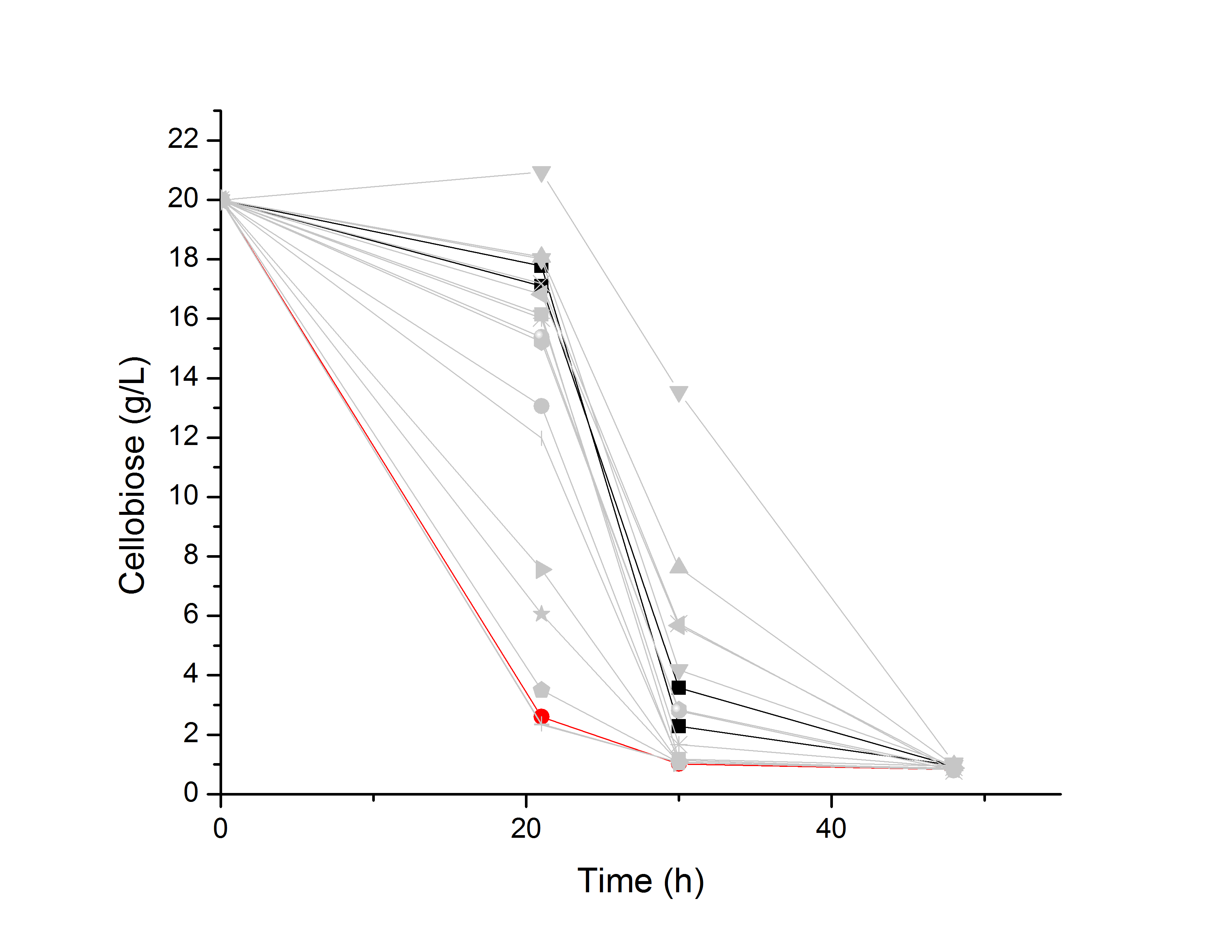


(b)

**Figure S3:** Glucose accumulation in the supernatant of the oxygen-limited fermentation. Errors derived from standard deviation of biological triplicates. (■) wild-type pathway, (●) R1 pathway, (▲) R2 pathway. The glucose is produced once most of the cellobiose has already been consumed.


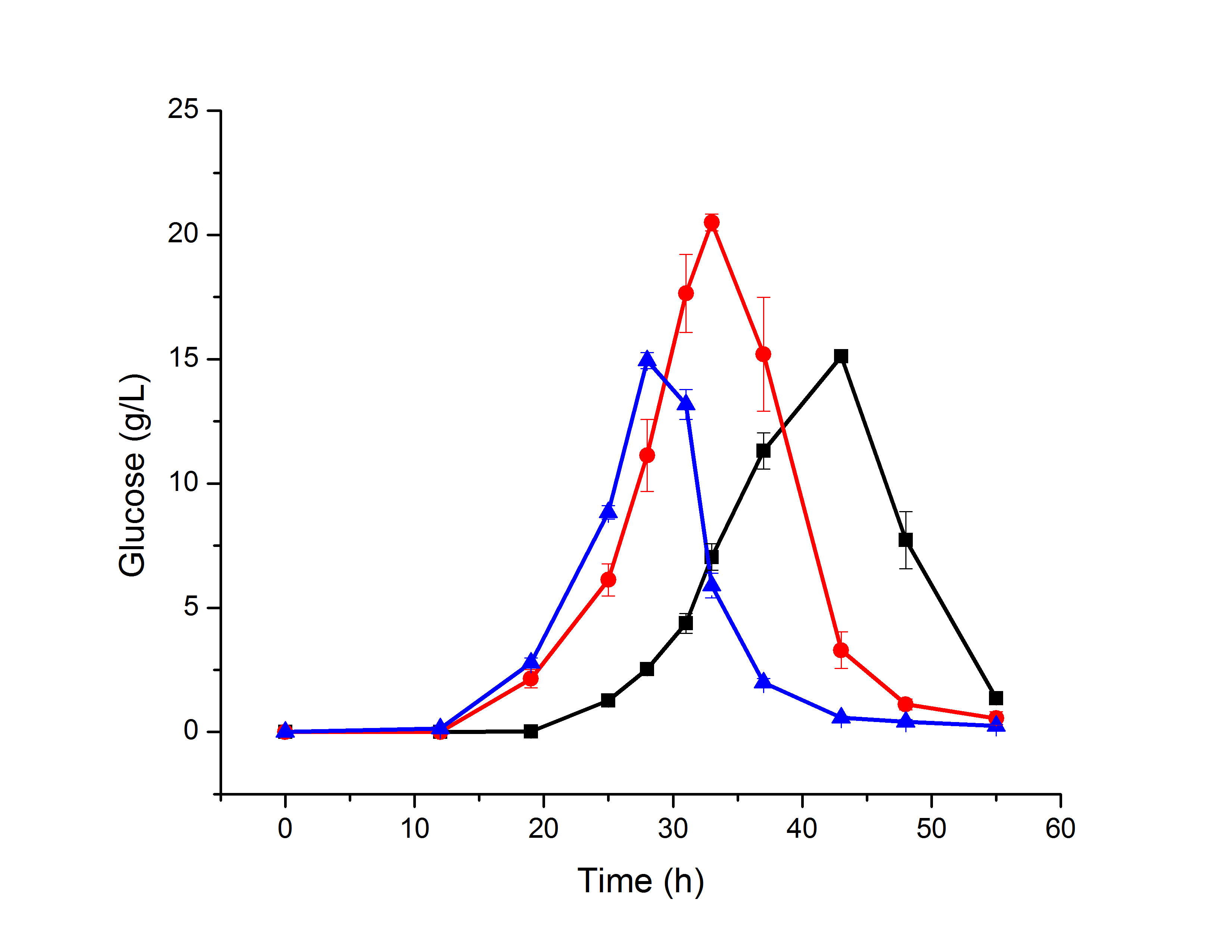


**Figure S4:** Consistent protein expression levels between mutants. Quantitative PCR analysis of relative mRNA levels of the mutations (a) of the *gh1-1*  and (b) the *cdt-1*. Relative expression is normalized to the average value of the housekeeping gene. GFP-fusion to the wild-type and mutant proteins was also performed to compare specific protein concentration of the strains (c) β-glucosidase and (d) cellodextrin transporter. Arbitrary fluorescence units (AFU) are normalized to the wild-type average value. Error bars represent the standard deviation of at least three biological triplicates. A two-tailed student *t*-test confirmed that there were no significant differences in mRNA levels or protein concentration levels when tagged with GFP (*p*-values all greater than 0.05).

(a)

(b)

(a)


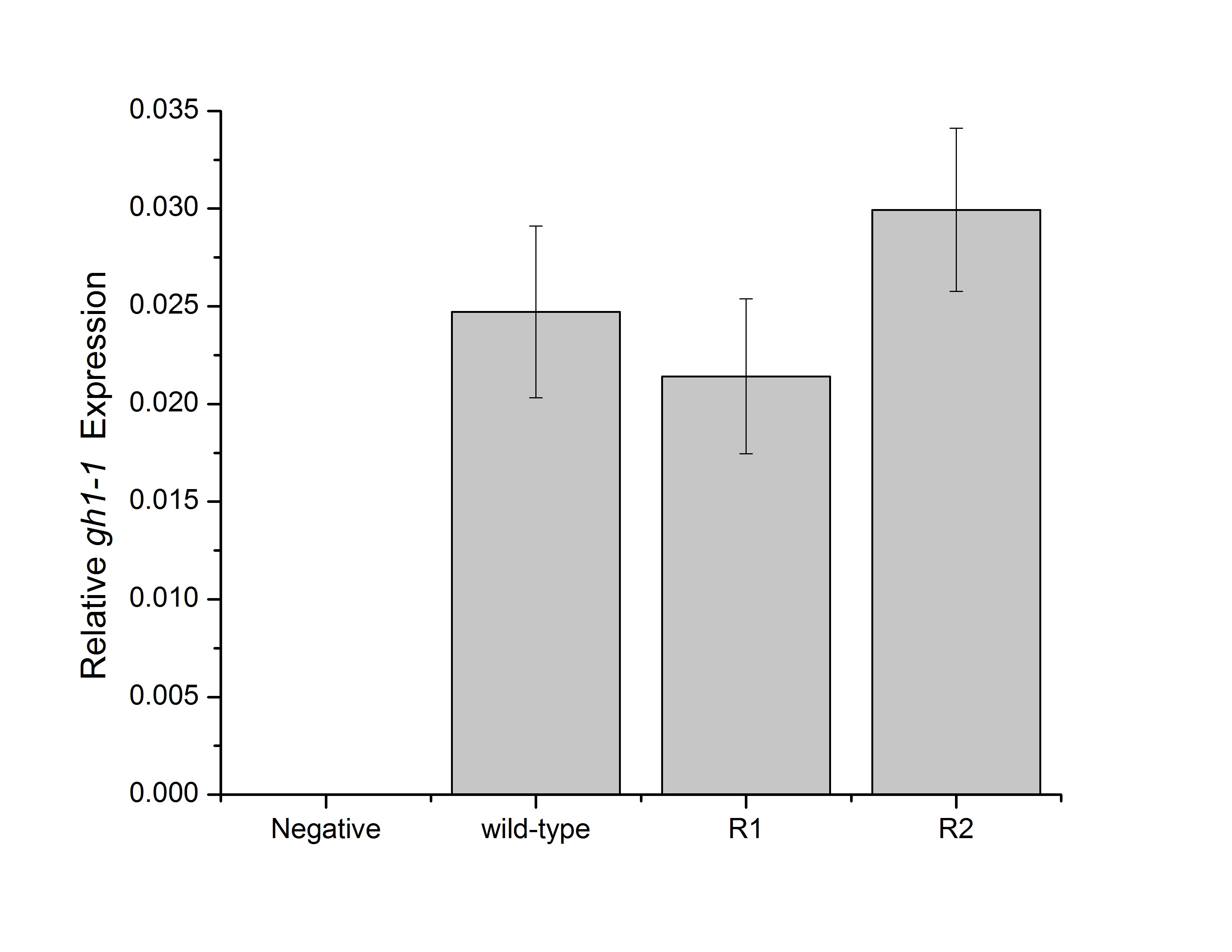

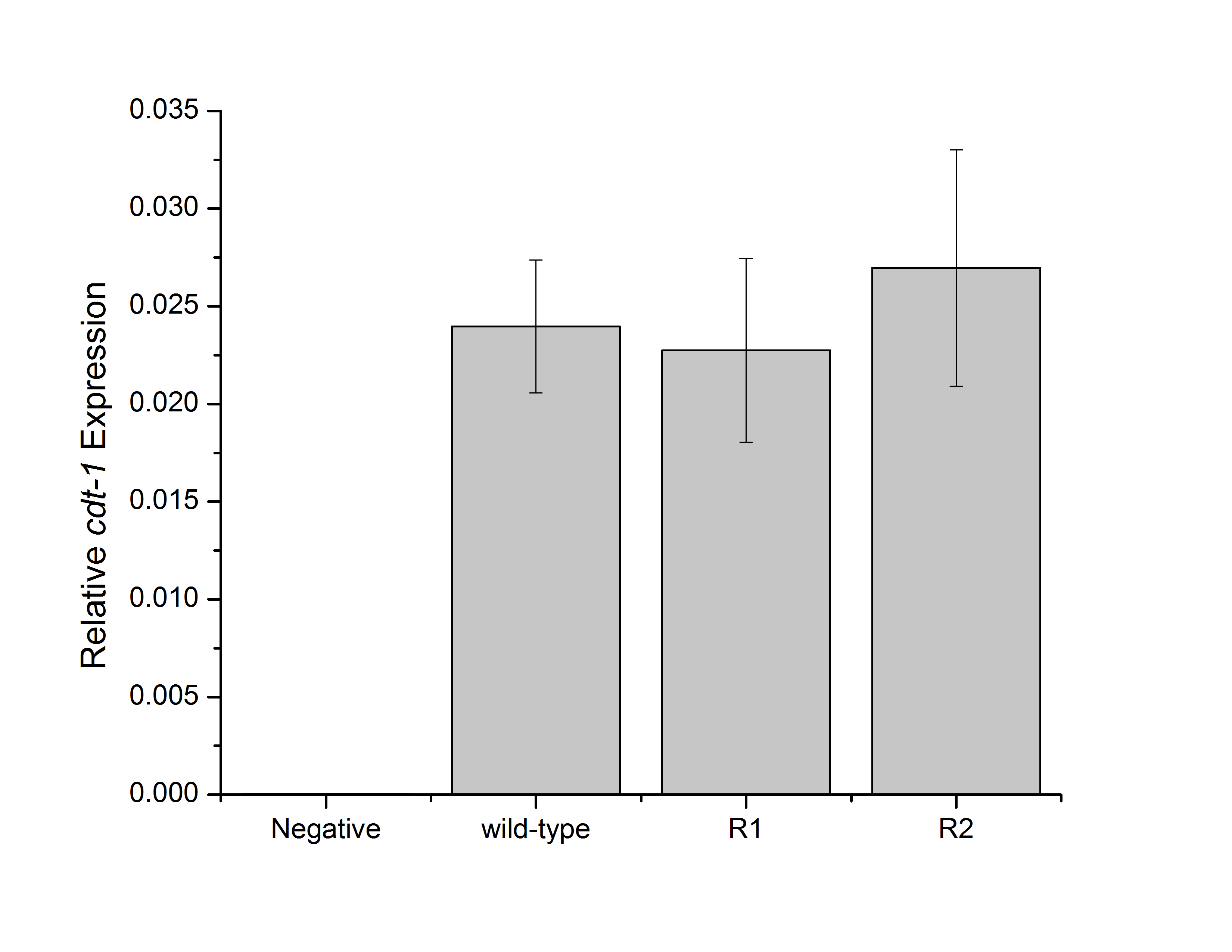


(c) (d)


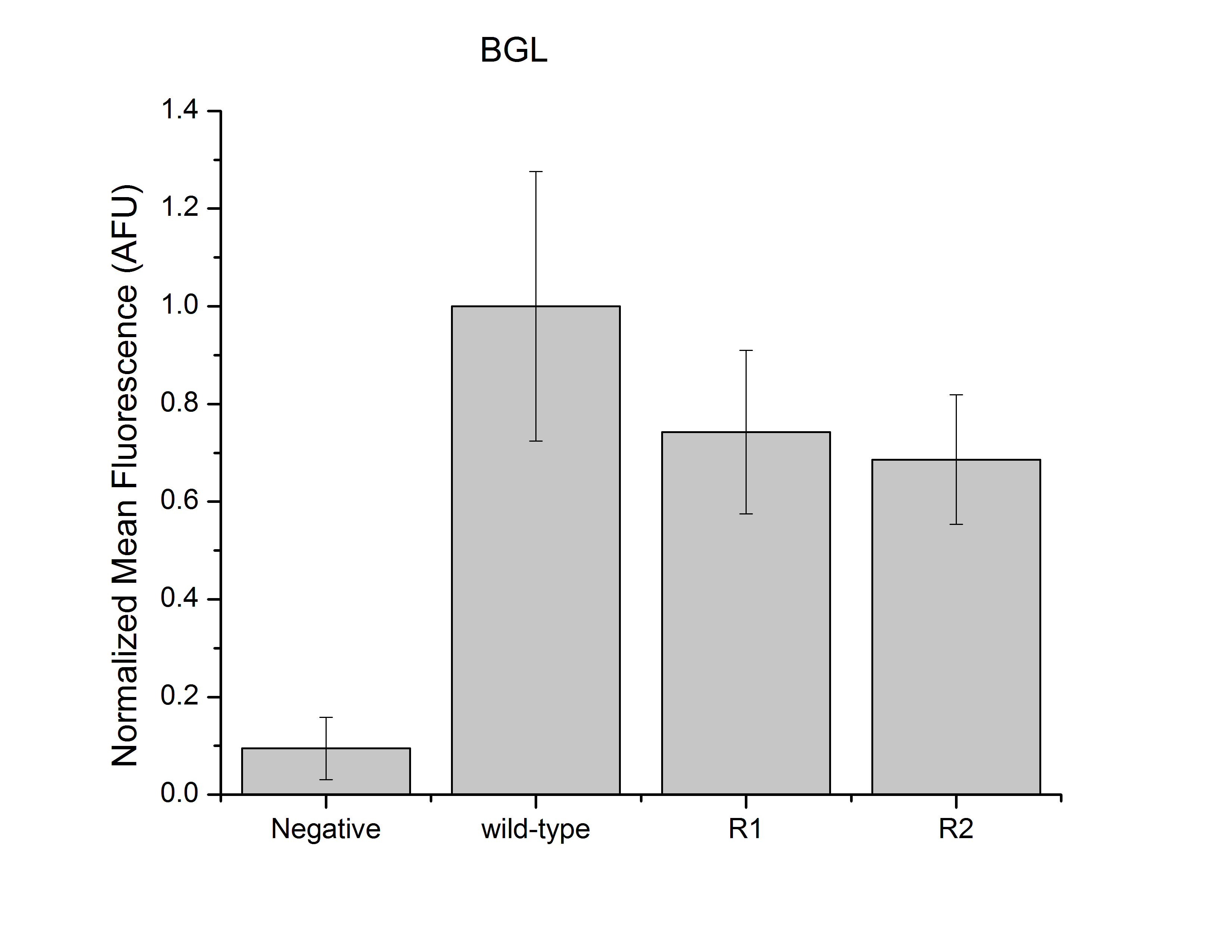

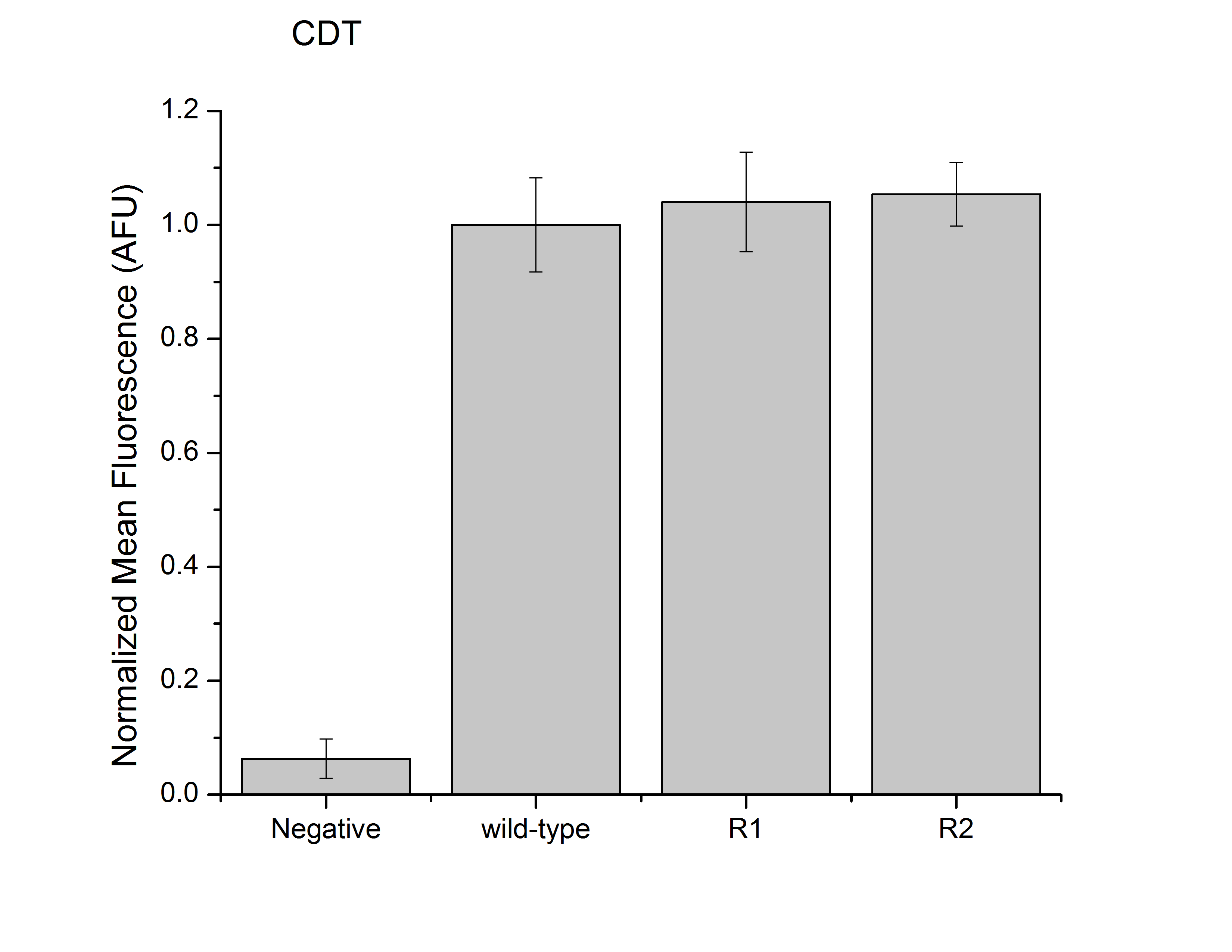

Supplement: Additional file 1: Table S1 — Primers used in this study. Figure S1. Selection plates of the error-prone library on cellobiose plates, depicting the large colonies. Figure S2. Flask screening data before retransformation of top clones from the first round of error-prone PCR. Figure S3. Flask screening data before retransformation of top clones from the first round of error-prone PCR. Figure S4. Consistent protein expression levels between mutants. [file 1475-2859-12-61-S1.docx]
